# Supplementary material for: Non-Invasive Early Detection of Oral Cancers Using Fluorescence Visualization with Optical Instruments
Source: Cancers (Basel). 2020 Sep 27;12(10):2771. doi: 10.3390/cancers12102771 (PMC7601016; doi:10.3390/cancers12102771)
Supplement: Supplementary file 1 [file cancers-12-02771-s001.pdf]

cancers

Supplementary Materials: Non-Invasive Early Detection of Oral Cancers Using Fluorescence Visualization with Optical Instruments

Takamichi Morikawa, Takahiko Shibahara, Takeshi Nomura, Akira Katakura, and Masayuki Takano

Figure S1. ROC for detection oral cancers

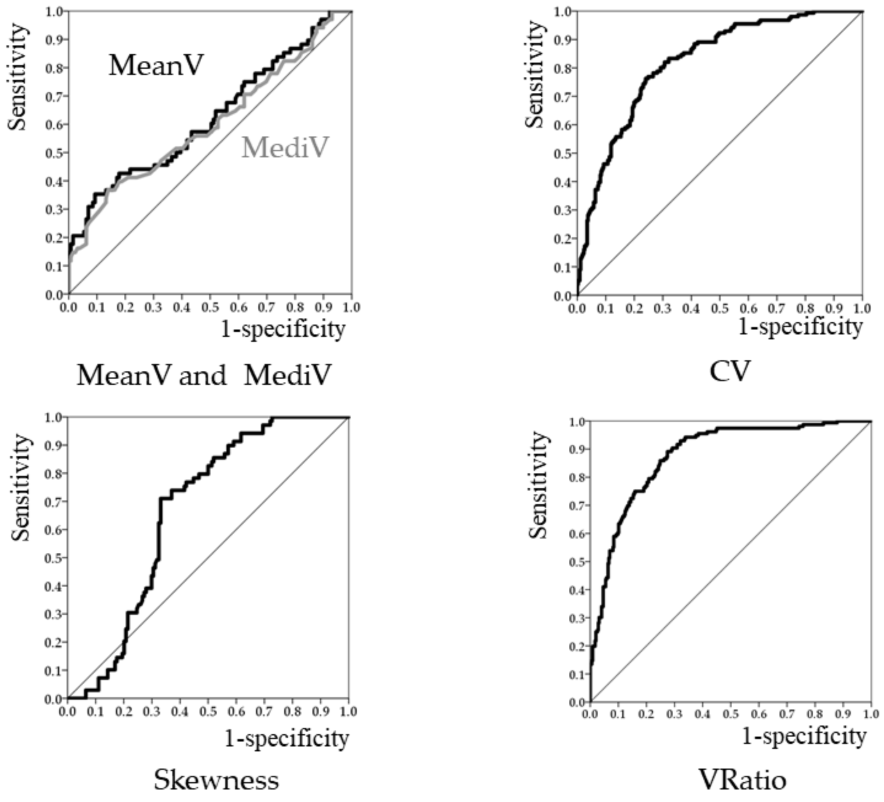

Figure 1. ROC for the detection of oral cancers.

Figure S2. Combination of each factors for detection of oral cancers

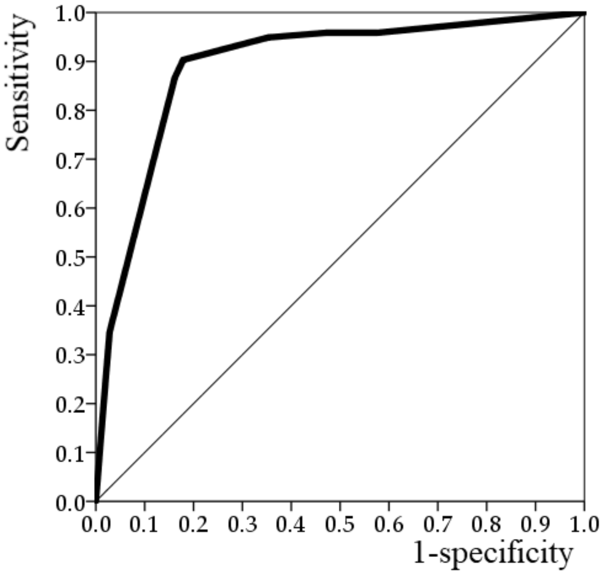

AUC	*p* value	Cut-off	Sensitivity	Specificity	95% CI
0.891	0.066	19.4	86.6	84.6	0.861, 0.921

Figure 2. Combination of each factor for the detection of oral cancers.

Figure S3. ROC for detection OPMDs

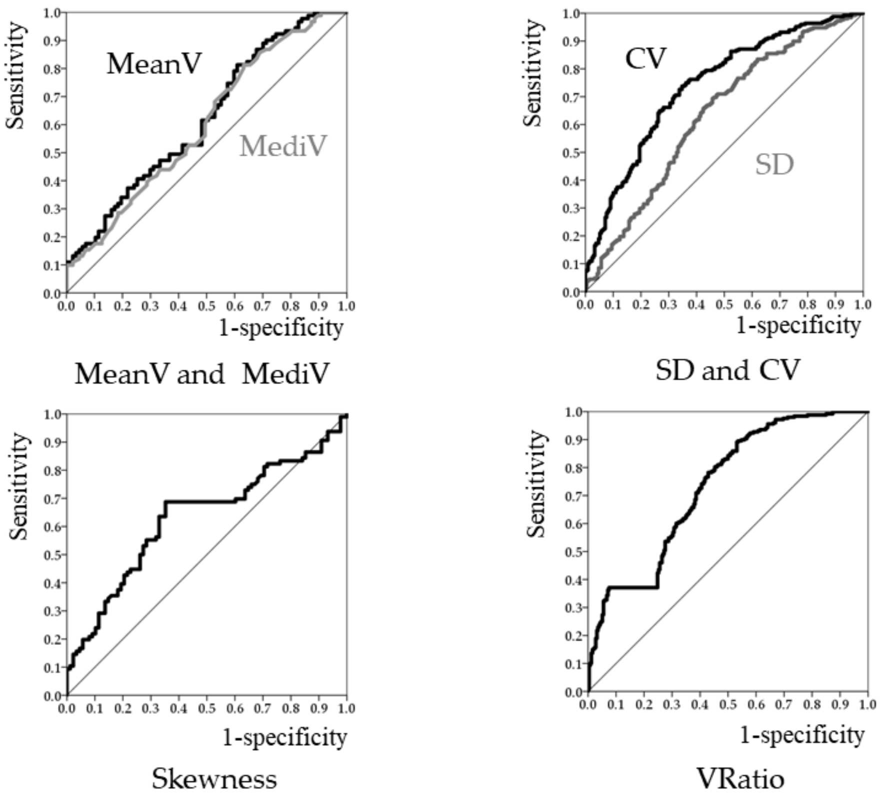

Figure 3. ROC for detection OPMDs.

Table 1. Patients characteristics.

Table S1. Patients characteristics

	n = 502
Sex, men/women	276 / 226
Age, mean	62.3
Site	
Tongue	259
Buccal mucosa	124
Gingiva	73
Plate	33
Others	13
Condition	
Oral squamous cell carcinoma	149
Carcinoma in situ	12
Oral lichen planus	123
Leukoplakia	102
Erythroplakia	3
Chronic candida	7
Stomatitis	32
Benign tumor	24
Normal mucosa	50
Control site, mean	
Area, (pixels)	944
MeanV	81.7
MediV	80.2
SD	2.9
CV	0.04
Skewness	45.3
kurtosis	-601
Lesion site, mean	
FVL rate, (%)	66.7%
Area, (pixels)	165,057
MeanV	66.3
MediV	62.8
SD	10.3
CV	0.16
Skewness	0.56
Kurtosis	0.48
VRatio (%)	85.2%

© 2020 by the authors. Licensee MDPI, Basel, Switzerland. This article is an open access article distributed under the terms and conditions of the Creative Commons Attribution (CC BY) license (http://creativecommons.org/licenses/by/4.0/).
